# MINDY1 promotes bladder cancer progression by stabilizing YAP

**DOI:** 10.1186/s12935-021-02095-4

**Published:** 2021-07-27

**Authors:** Yongwen Luo, Jun Zhou, Jianing Tang, Fengfang Zhou, Zhiwen He, Tongzu Liu, Tao Liu

**Affiliations:** 1 Department of Urology, Zhongnan Hospital of Wuhan University, Wuhan, China; 2 Department of Biological Repositories, Zhongnan Hospital of Wuhan University, Wuhan, China; 3 The Interventional Diagnosis and Treatment Center, Zhongnan Hospital of Wuhan University, Wuhan, China

**Keywords:** Bladder cancer, MINDY1, YAP, Stabilization, Ubiquitination

## Abstract

**Background:**

Bladder cancer is one of the most commonly diagnosed urological malignant tumor. The Hippo tumor suppressor pathway is highly conserved in mammals and plays an important role in carcinogenesis. YAP is one of major key effectors of the Hippo pathway. However, the mechanism supporting abnormal YAP expression in bladder cancer remains to be characterized.

**Methods:**

Western blot was used to measure the expression of MINDY1 and YAP, while the YAP target genes were measured by real-time PCR. CCK8 assay was used to detect the cell viability. The xeno-graft tumor model was used for in vivo study. Protein stability assay was used to detect YAP protein degradation. Immuno-precipitation assay was used to detect the interaction domain between MINDY1 and YAP. The ubiquitin-based Immuno-precipitation assays were used to detect the specific ubiquitination manner happened on YAP.

**Results:**

In the present study, we identified MINDY1, a DUB enzyme in the motif interacting with ubiquitin-containing novel DUB family, as a bona fide deubiquitylase of YAP in bladder cancer. MINDY1 was shown to interact with, deubiquitylate, and stabilize YAP in a deubiquitylation activity-dependent manner. MINDY1 depletion significantly decreased bladder cancer cell proliferation. The effects induced by MINDY1 depletion could be rescued by further YAP overexpression. Depletion of MINDY1 decreased the YAP protein level and the expression of YAP/TEAD target genes in bladder cancer, including CTGF, ANKRD1 and CYR61.

**Conclusion:**

In general, our findings establish a previously undocumented catalytic role for MINDY1 as a deubiquitinating enzyme of YAP and provides a possible target for the therapy of bladder cancer.

**Supplementary Information:**

The online version contains supplementary material available at 10.1186/s12935-021-02095-4.

## Background

Bladder cancer is one of the most commonly diagnosed urological malignant tumor with annually increasing global incidence [1]. Approximately 75% of bladder cancer cases are initially diagnosed as non-muscle-invasive bladder cancer (NMIBC). Unfortunately, 25% of tumors have already infiltrated the bladder muscle wall and are classified as muscle‐invasive bladder cancer (MIBC) at the initial diagnosis [2]. It is believed that NMIBC is originated from simple hyperplasia and minimal dysplasia. NMIBS seems to have high genomic stability, and have a relative low risk of metastasis. MIBC is originated from flat dysplasia and carcinoma in situ, which is genetically unstable and has a high risk of metastasis [3]. The standard treatment strategy for patients with NMIBC is transurethral resection of tumor and intravesical chemotherapy, while the five-year recurrence can add up to 31–78% and 10–30% of patients ultimately lead to invasive tumors after management [4]. For MIBC, radical cystectomy with platinum-based chemotherapy regimens is considered as the optimal treatment strategy. While the majority of the patients till have poor outcomes after systemic therapy [5, 6]. Recent advances of genome sequencing and bioinformatics have provided numerous molecular biomarkers to guide the treatment planning of bladder cancer patients, while only a few have been validated for clinical use or recommended by guidelines. Therefore, it is important to find an effective therapeutic strategy to cure or to prolong the survival of patients with bladder cancer.

The Hippo pathway is highly conserved in mammals which was initially identified from Drosophila [7]. WW domain-containing transcription factor (WWTR1 or TAZ) and Yes-associated protein (YAP) are the two major downstream effectors. As transcriptional co-activators, YAP and TAZ mediate the biological functions of the Hippo pathway by regulating gene transcription [8]. The activity of YAP and TAZ is mainly regulated by the MST1/2-Lats1/2 kinase cascade by directly phosphorylating YAP/TAZ on multiple sites, resulting in interaction with 14-3-3 protein and cytoplasmic retention. When Hippo signaling is off, YAP/TAZ enter the nucleus, and recruit other factors, such as TEAD and RUNX to activate genes involved in cell proliferation, migration, survival and metabolism [9–11]. The dysregulation of Hippo pathway is thought to play a crucial role during tumor invasion and metastasis. YAP and TAZ are frequently activated in a variety of human malignancies. The activation of YAP/TAZ can promote cancer cell proliferation, metastasis, chemoresistance, and cancer stem cell-features, making them promising therapeutic targets in cancer [12]. While the underlying mechanisms regarding YAP/TAZ activation or overexpression in malignant tumors have not been well defined.

Ubiquitination is a post-translational modification that is essential for cellular homeostatic maintenance [13]. Accumulating evidence has indicated that ubiquitination is involved in processes such as cell-cycle progression, cell survival, apoptosis, DNA repair, and antigen presentation [14]. Accumulating studies indicate that the Hippo pathway is tightly modulated by the ubiquitin–proteasome system. A number of E3 ligases, such as PRAJA, ITCH, SIAH2, FBW7, and WWP1 are shown to play an essential role in controlling the abundance of several Hippo pathway components [15–17]. For instance, Fbxw7 regulates YAP protein stability by targeting YAP for ubiquitination and proteasomal degradation in hepatocellular carcinoma [16]. Deubiquitinases (DUBs) can reverse the ubiquitination of proteins by removing ubiquitin from the substrates. The DUBs in the human genome can be categorized into six families: ubiquitin COOH-terminal hydrolases (UCH), ubiquitin-specific proteases (USP), the JAB1/MPN/MOV34 family (JAMM), Josephins, ovarian tumor proteases (OTU), and motif interacting with ubiquitin-containing novel DUB family (MINDY) [18]. However, how DUBs regulate the Hippo signaling in bladder cancer remains less well understood.

In the present study, we found that MINDY1 was a possible deubiquitinase responsible for YAP deubiquitination and stabilization in bladder cancer. We also found that MINDY1 promoted cell proliferation through YAP. Overall, our study has demonstrated that MINDY1 is a novel deubiquitinating enzyme of YAP and may prove to be a potential target for bladder cancer intervention.

## Materials and methods

### Cell culture

The human bladder cancer cell lines T24, UMUC3 and human embryonic kidney HEK293 cells were purchased from American Type Culture Collection (ATCC). T24 cells were cultured with RPMI-1640 (42,401, Life Technologies) supplemented with 10% fetal bovine serum (FBS, Gibco, Life Technologies, 10,270). UMUC3 and HEK293 were culture with Dulbecco’s Modified Eagle’s Medium (DMEM) that contains 4 mM L-glutamine and 4,5 g/L glucose (41,965, Life Technologies) supplemented with 10% FBS All cells were cultured at 37 °C in an atmosphere of 5% CO_2_.

### Plasmids and RNA inference

Wild type (WT) MINDY1 and its deletion mutant plasmids were obtained from Hanbio Biotechnology Co., Ltd. (Shanghai, China). The HA-K6, -K11, -K27, -K29, -K33, -K48, -K63, and -Ub plasmids were acquired from Addgene. Small interfering RNAs targeting MINDY1(siG000055793A-1-5, siG000055793B-1-5) were obtained from Ruibo Biotechnology Co., Ltd. (Guangzhou, China). The YAP full- and its deletion constructs were gifted from Dr. Gaosong Wu and were described in the previous study [19]. Lipofectamine 2000 (Invitrogen, Carlsbad, CA, USA) was used for plasmid transfection according to the manufacturer’s instructions.

### RNA extraction and qPCR analysis

Total RNA was extracted from the cancer cells using the RNeasy plus mini kits (Qiagen, Germany) following manufacturer’s instructions. Reverse transcription was performed using the PrimeScript RT Master Mix (Takara, Japan). qRT-PCR was performed using the SYBR green mix (Toyobo, Japan) with the CFX96TM Real-time PCR Detection System (Bio-Rad, USA) normalized to GAPDH. The 2^−ΔΔCt^ method was used to calculate the relative expression. All assays were performed in triplicates. Primers were listed as follows: GAPDH (forward: 5′-ACGGGAAGCTTGTCATCAAT-3′, reverse: 5′-TGGACTCCACGACGTACTCA-3′); YAP (forward: 5′-TAGCCCTGCGTAGCCAGTTA-3′; reverse: 5′- TCATGCTTAGTCCACTGTCTGT-3′).

### Cell proliferation analysis

The cell proliferation rate was detected using Cell Counting Kit-8 (CCK8) assay at indicated time points according to the manufacturer's instructions. T24 and UMUC3 cells were transfected with the indicated siRNA, and 24 h later, cells were digested and 2 × 10^3^ cells were seeded in 96-well culture plates. CCK8 solution reagent was added to each well and incubated for 1.5 h at 37 °C. The absorbance was measured at 450 nm using a microplate reader. For clone formation assay, cells were digested and seeded into 6-well plates at a density of 1000 cells per well. After 14-day incubation, cells were fixed with 4% paraformaldehyde and visualized by 0.5% crystal violet staining. EdU incorporation assay was performed using Cell-LightTM EdU Apollo 567 In Vitro Kit (Cat number: C10310-1, RiboBio, Guangzhou, China) according to the manufacturer’s protocol, and images were captured using an Olympus microscope.

### In vivo tumorigenesis assay

BALB/c nude mice aged 4 weeks were obtained from Beijing HFK Bioscience Co., Ltd. in Beijing, China. For in vivo tumorigenic experiment, 1 × 10^6^ T24 cells were injected to the right dorsal flank of each mouse. Tumor sizes were measured every 5 days until the end of the experiment. The mice were maintained in a temperature and humidity‐controlled and specific pathogen‐free environment in the laboratory animal facility of Zhongnan Hospital of Wuhan University. The experiments were performed under the protocols approved by ethnic committee of Zhongnan Hospital of Wuhan University.

### Luciferase assay

The YAP/TEAD luciferase reporter plasmid was transfected into T24 cells together with the Renilla plasmid. After 24 h, luciferase activity of YAP/TEAD luciferase reporter was measured using the dual-Luciferase Reporter kit (Promega, Germany) following the manufacturer's protocol.

### Co-immunoprecipitation assay

Cells were lysed with NP-40 lysis buffer containing a cocktail of protease inhibitors. The total cell lysis was precleared with rabbit IgG for 2 h and subsequently immunoprecipitated with the indicated antibody at 4 °C overnight. Protein A/G PLUS-Agarose beads (Santa Cruz) were then added to the lysates and incubated at 4 °C for 2 h. The immunocomplexes were washed with lysis buffer three times and separated by SDS-PAGE. Immunoblotting was performed following standard procedures.

### Western blot analysis

Cells were lysed with RIPA extraction reagent (Beyotime, China) supplemented with protease inhibitors (Sigma-Aldrich, USA). Total protein was separated using 10–12.5% sodium dodecyl sulfate polyacrylamide gel electrophoresis and transferred to 0.45 μm PVDF membrane (Millipore, USA). Primary antibodies were YAP (Proteintech, 13584-1-AP), MINDY1 (Invitrogen, PA5-55825), HA (Proteintech, 51064-2-AP), Myc (Proteintech, 60003-2-Ig), and GAPDH (Proteintech, 60004-1-Ig) antibodies. Bands were visualized using an enhanced chemiluminescence (ECL) kit (Boster, China) and detected by ChemiDoc XRS + Imaging System (Bio-Rad).

### Tissue microarray and immunohistochemistry

Commercially available tissue microarray (TMA) slides (Alenabio, China) were purchased for immunohistochemistry (IHC) analysis. Specific primary antibodies against MINDY1 (abcam, USA) and YAP (Proteintech, China) were used for IHC. Whole slide image capture was performed on the EVOS auto cell image system (Life technology, USA). The immunohistochemical score were assessed by two experienced pathologists without knowledge of patients’ characteristics. Scores were calculated on intensity and percentage of positive staining tumor cell nuclei or cytoplasm in the whole tissue stains were evaluated according to Fromowitz Standard. Briefly, the staining intensity was graded as follows: no staining, 0; weakly positive, 1; moderately positive, 2; and strongly positive, 3. The percentage of positive cells was into four grades: 0–25% staining, 1; 26–50% staining, 2; 51–75% staining, 3; and 76–100% staining, 4. The multiplication of the intensity and percentage scores was used to calculate the final staining score. For quantification, all stains were assessed at 200× magnifications and at least three fields from each core were counted. MINDY1 expression was qualified as low (*IHC* *score* 0–3) and high (*IHC* *score* 4–12).

### Ethical statement

The research was carried out according to the World Medical Association Declaration of Helsinki and was approved by the Ethics Committee at Zhongnan Hospital of Wuhan University.

### Statistical analysis

Student’s t test and one-way ANOVA were used to compare two and more groups respectively. Multiple comparison with Bonferroni correction was performed when appropriate. A P value < 0.05 was considered as statistically significant and all tests were two-tailed. All statistical tests were performed with Prism 7.0 (GraphPad, USA).

## Results

### MINDY1 depletion inhibits Hippo signaling pathway activity

**Fig. 1:**
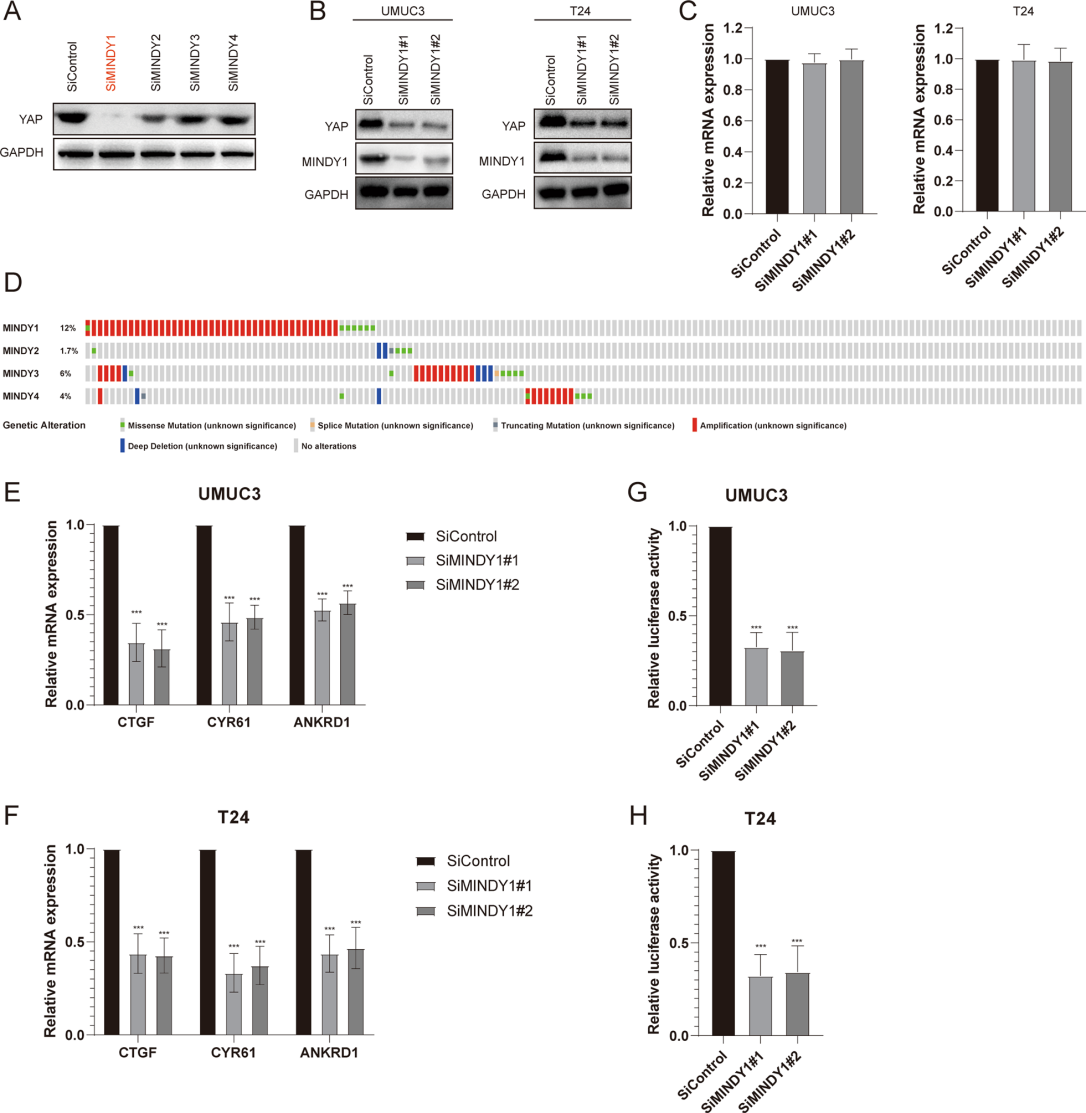
MINDY1 depletion decreases Hippo signaling activity in bladder cancer cells. **A** The siRNAs specific to each deubiquitinating enzyme (MINDY1-4) were transfected into T24 cells. After 48 h, cells were lysed and the YAP protein level was analyzed by Western blot. Relative YAP protein level was normalized to GAPDH. **B** MINDY1 depletion decreased YAP protein level. **C** MINDY1 depletion did not affect YAP mRNA level. **D** Genetic alternations of MINDYs in bladder cancer. **E**, **F** MINDY1 depletion decreased YAP target genes. Bladder cancer cells were transfected with si MINDY1 or siControl. Total RNA was prepared and the expression of the endogenous YAP target genes, CTGF, CYR61 and ANKRD1 were determined by qRT-PCR. **G**, **H** MINDY1 depletion affected YAP/TEAD-luciferase activity. Bladder cancer cells were transfected with siMINDY1 or siControl together with YAP/TEAD luciferase reporter plasmid. Luciferase activity was measured 48 h after transfection. *, *P value* < 0.05; **, P value < 0.01; ***, P value < *0.001*

To identify the motif interacting with Ub-containing novel DUB family (MINDY) responsible for YAP deubiquitination and stabilization in bladder cancer. Four nonoverlapping siRNA mixtures specific for each of the DUBs belonging to MINDY were transfected into T24 cells, it was found that silencing MINDY1 markedly decreased YAP (Fig. 1A). In addition, we detected the MINDY1 protein levels in four bladder cancer cell lines (5637, UM-UC-3, T24 and BIU-87) and immortalized normal uroepithelial cell line (SV-HUC-1), exhibiting an upregulation tendency of MINDY1 in bladder cancer cell lines (Additional file 1: Figure S1). We then depleted MINDY1 with two non-overlapping siRNAs separately in T24 and UMUC3 cells to further validate the function of MINDY1 in regulating YAP, as shown in Fig. 1B, C, MINDY1 depletion significantly decreased the YAP protein levels without influence on the expression of YAP mRNA. Genomic analysis of all the MINDYs in human bladder cancer samples revealed MINDY1 amplification was observed in 12% of cases (Fig. 1D). We then examined the expression of YAP target genes (CTGF, CYR61 and ANKRD1) and found that depletion of MINDY1 dramatically decreased the transcripts of CTGF, CYR61 and ANKRD1 (Fig. 1E, F). In addition, we measured the YAP/TEAD-luciferase reporter gene activity by MINDY1 depletion to determine whether MINDY1 depletion affected the YAP transcriptional activity. It was found that depletion of MINDY1 decreased the YAP/TEAD-luciferase reporter gene activity (Fig. 1G, H). All these results demonstrated that MINDY1 was a regulator of the Hippo signaling pathway.

### MINDY1 interacts with YAP

**Fig. 2:**
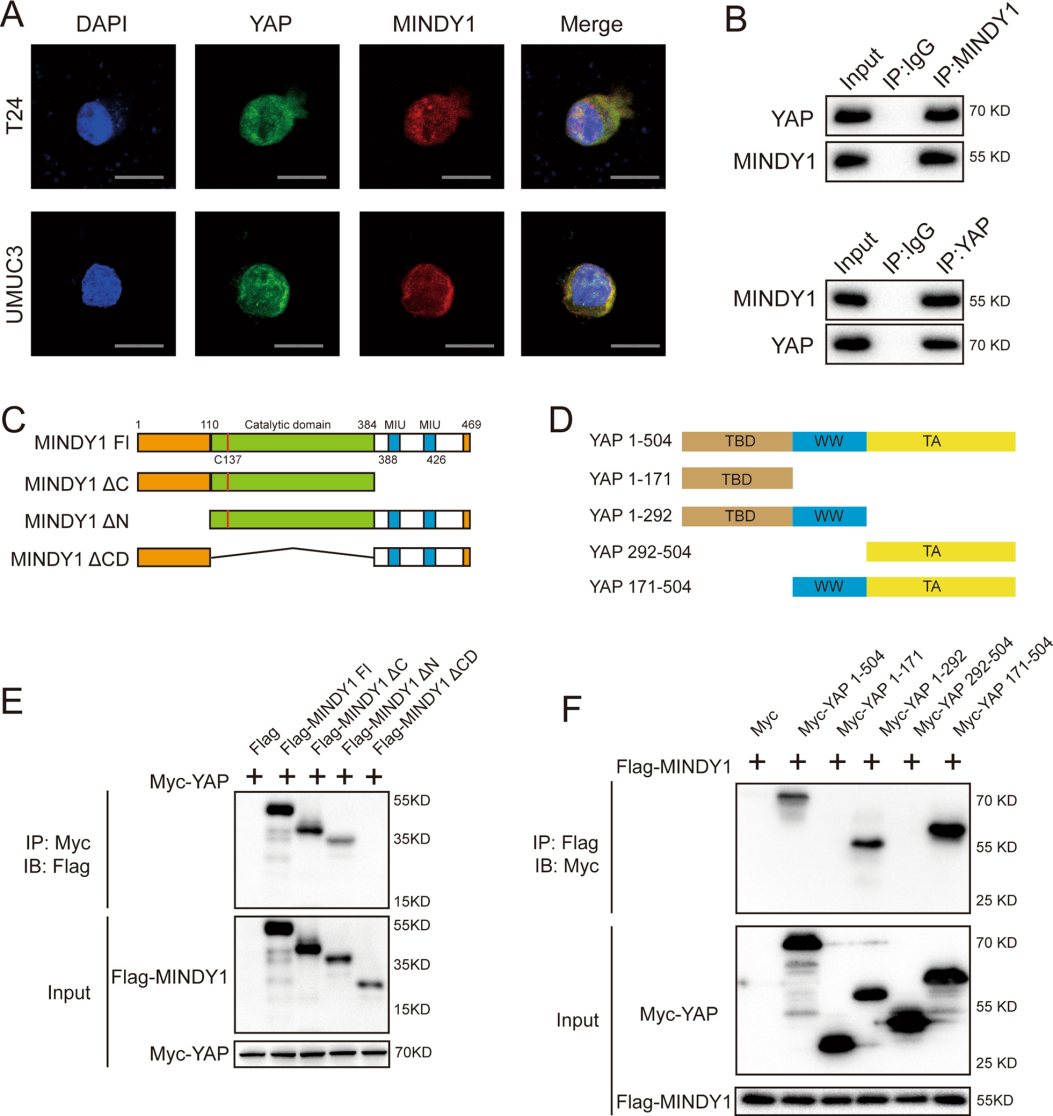
MINDY1 associates with YAP and increases its stability. **A** An immunofluorescence assay demonstrated that MINDY1 and YAP at least partially colocalized in T24 and UMUC3 cells. **B** Co-IP assay revealed an association between endogenous MINDY1 and YAP in T24 cells. T24 cells were harvested with RIPA lysis buffer. Co-IP was performed using antibody as indicated. **C**, **D** MINDY1 and YAP domain structure and deletion mutants used in the study. **E** The catalytic domain of MINDY1 interacted with YAP. HEK293 cells were transfected with 2 µg Myc-YAP together with Flag-MINDY1 full length or mutants. After 24 h, cells were harvested with NP-40 lysis buffer. Co-IP was performed using Myc antibody. The possible interacted MINDY1 domains were detected by Flag antibody. **F** YAP interacted with MINDY1 through its WW terminal. HEK293 cells were transfected with 2 µg Flag-MINDY1 together with Myc-YAP full length or mutants. After 24 h, cells were harvested with NP-40 lysis buffer. Co-IP was performed using Flag antibody. The possible interacted YAP domains were detected by Myc antibody

Results of immunostaining demonstrated that YAP and MINDY1 colocalized both in the nucleus of bladder cancer cells (Fig. 2A). We found that endogenous MINDY1 coimmunoprecipitated with endogenous YAP in the co-immunoprecipitation (Co-IP) experiment (Fig. 2B). YAP has three functional domains: TEAD transcription factor-binding domain (TBD), WW domain, trans-activation domain (TAD). MINDY1 has a catalytic domain which cleaves K48 chains (Fig. 2C, D). We then made these deletion constructs in order to delineate the interaction between YAP and MINDY1. The full length of MINDY1 or MINDY1 deletion constructs (ΔC terminal, ΔN terminal, ΔCatalytic domain) was expressed together with YAP in HEK293 cells. Co-IP assay indicated that catalytic domain (110–384) was required for MINDY1 to interact with YAP (Fig. 2E). On the other hand, the full length of YAP or deletion constructs (ΔTBD, ΔTA, ΔTBD + WW and ΔWW + TA) was expressed together with MINDY1 in HEK293 cells. Co-IP assay showed that WW domain of YAP was necessary for its interaction with MINDY1 (Fig. 2F).

### MINDY1 increases the stability of YAP

**Fig. 3:**
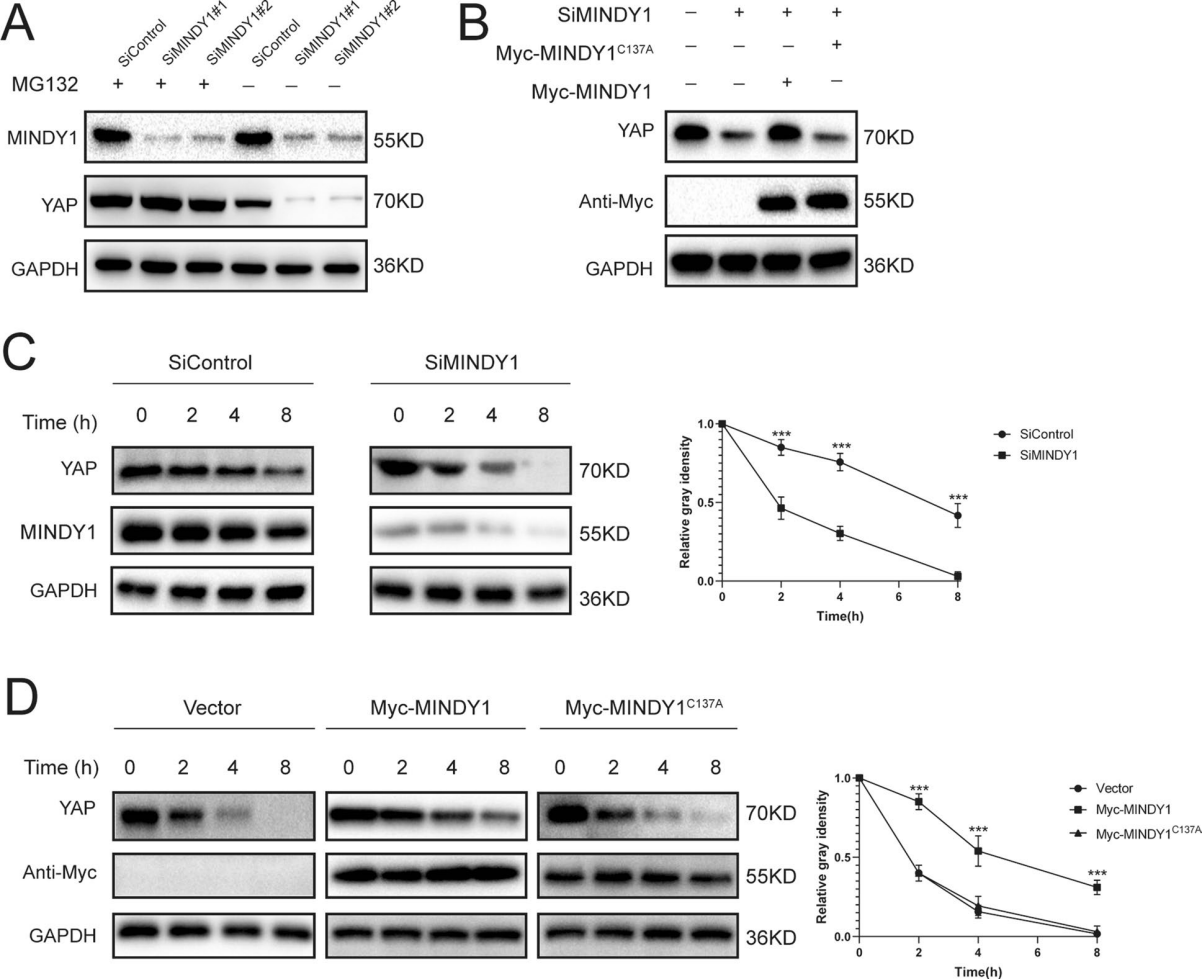
MINDY1 increases YAP stability. **A** In the presence of the proteasome inhibitor MG132, depletion of MINDY1 did not further decrease the YAP protein level. Bladder cancer cells were transfected with siMINDY1 or siControl. After 48 h, cells were treated with 10 µM MG132 / vehicle for 6 h, cell lysates were prepared for western blot analysis. **B** T24 cells were transfected with MINDY1 (wild type or C137A) together with MINDY1 siRNA. The YAP protein levels were measured. **C** MINDY1 depletion decreased YAP half-life in bladder cancer cells. Bladder cancer cells were transfected with siMINDY1 or siControl. After 48 h, cells were treated with 100 µM cycloheximide/vehicle for indicated times. Cell lysates were prepared for western blot analysis. **D** MINDY1^C137A^ did not increase YAP half-life in HEK293 cells. HEK293 cells were transfected with YAP plasmid and Myc-tag, Myc-MINDY1 or Myc- MINDY1^C137A^ plasmids. After 24 h, cells were treated with 100 µM cycloheximide/vehicle for indicated times. Cell lysates were prepared for Western blot analysis

The interaction between MINDY1 and YAP suggested that YAP might be a substrate of MINDY1, and therefore we evaluated the possibility of YAP deubiquitylation by MINDY1. It was found that MINDY1 deletion dramatically decreased YAP protein level, and this effect could be reversed by addition of the proteasome inhibitor MG132 or overexpression of MINDY1-WT, but not its catalytically inactive mutant MINDY1^C137A^ (Fig. 3A, B). We then treated cells with the protein synthesis inhibitor cycloheximide to prove that MINDY1 affected ERα stability. The stability of YAP was significantly decreased in cells depleted of MINDY1 (Fig. 3C). In cells overexpressing MINDY1-WT, but not MINDY1^C137A^, half-life of YAP was prolonged (Fig. 3D).

### MINDY1 deubiquitylates YAP

**Fig. 4:**
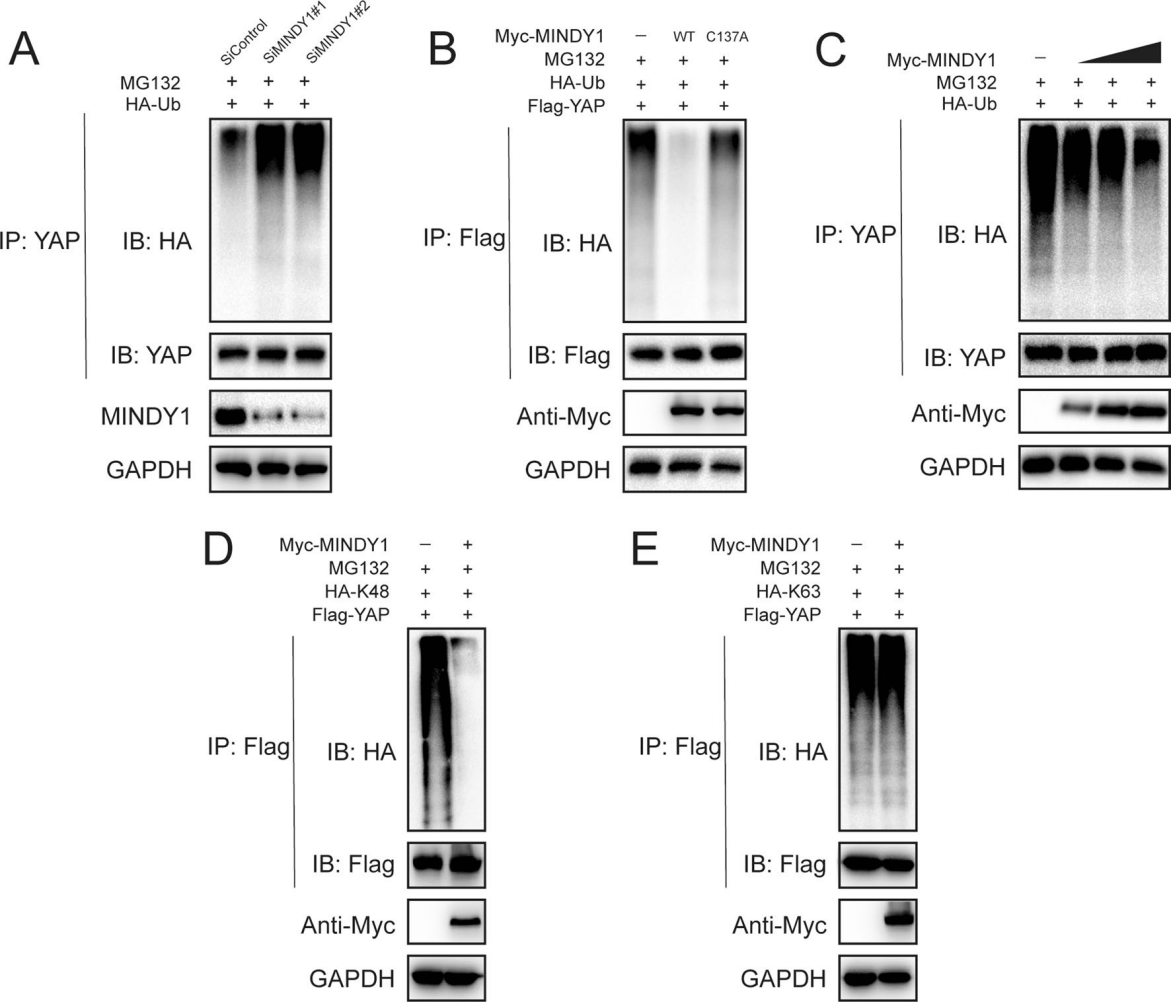
MINDY1 deubiquitylates YAP. **A** T24 cells transfected with the indicated siRNA were treated with MG132 for 6 h before collection. YAP was immunoprecipitated with anti-YAP and immunoblotted with anti-HA. **B** Immunoblotting to detect the ubiquitination of YAP in HEK293 cells co-transfected with Flag-YAP, HA-Ubiquitin and Myc-MINDY1 (wild type or C137A). **C** MINDY1 removed the ubiquitin chain of YAP in a dose-dependent manner. **D**, **E** K48 or K63 Ub was co-transfected with Flag-YAP and Myc- MINDY1 into HEK293 cells. After treatment with 10 μM MG132 for 6 h, cell lysates were subjected to ubiquitination assay and the ubiquitination level of YAP was detected by HA antibody

Depletion of MINDY1 significantly increased the level of ubiquitinated-YAP in T24 cells (Fig. 4A). Conversely, ectopic expression of MINDY1-WT, but not MINDY1^C137A^, markedly decreased YAP ubiquitylation in cells (Fig. 4B). In vivo deubiquitylation assays showed that MINDY1 directly removed the ubiquitin chain of YAP in a dose-dependent manner (Fig. 4C). Then, we examined MINDY1 deubiquitination activity on YAP in two common ubiquitination manners (K48-linked ubiquitination and K63-linked ubiquitination). Previous studies showed that K48-linked ubiquitination of YAP leaded to protein degradation, while K63-linked ubiquitination of YAP linked to non-proteolytic modification and promoted YAP co-activator function in the nuclear. It was found that MINDY1 efficiently removed the K48-linked ubiquitin chain on YAP (Fig. 4D, E). Taken together, MINDY1 was identified as a specific DUB, which de-polyubiquitylated and stabilized YAP.

### MINDY1 regulates cell proliferation

**Fig. 5:**
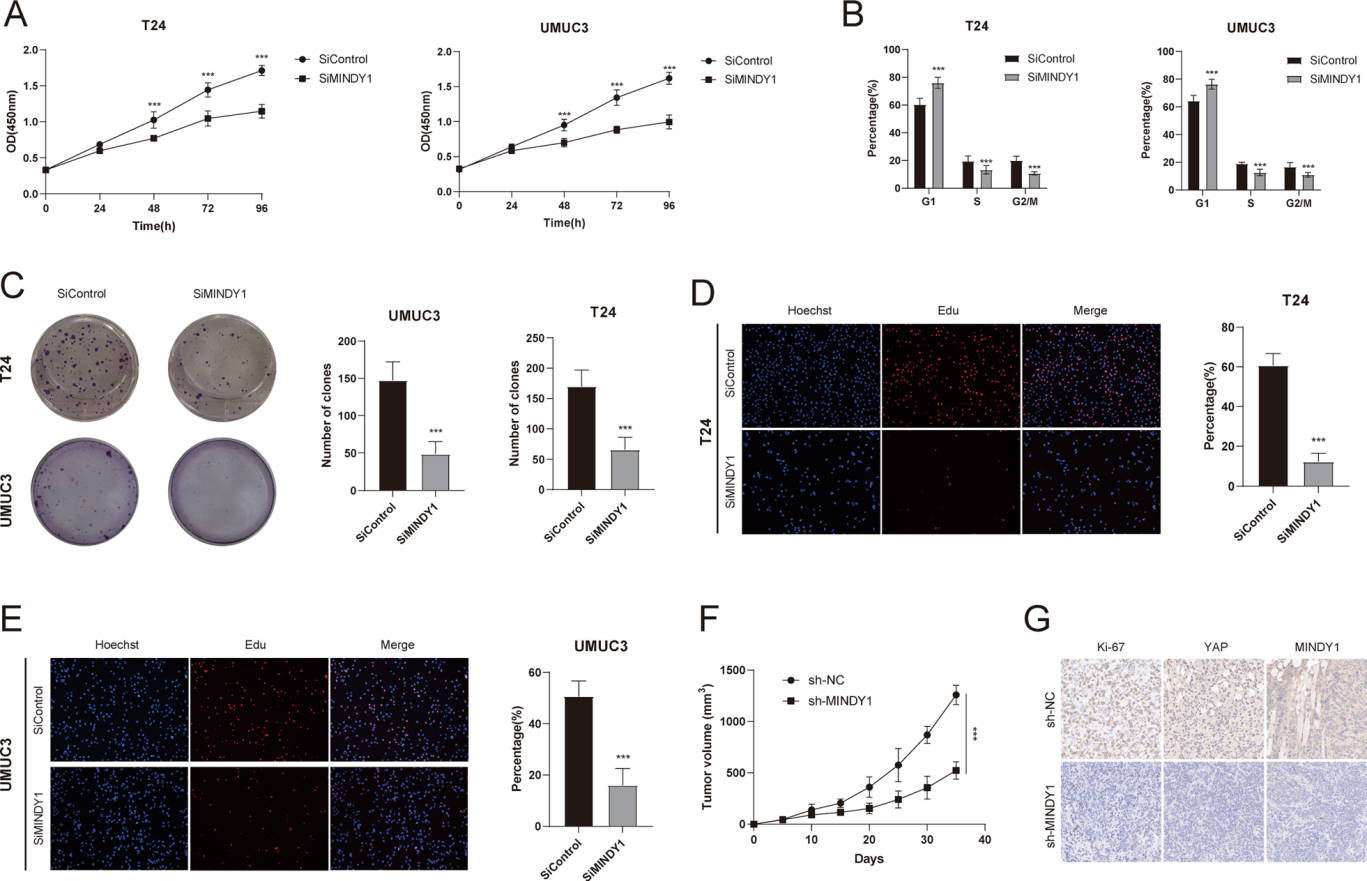
MINDY1 depletion inhibits bladder cancer cell proliferation. **A** MINDY1 depletion inhibited cell proliferation in bladder cancer cells. **B** MINDY1 depletion induced G1 cell cycle arrest in bladder cancer cells. **C** MINDY1 depletion decreased clone formation capability of bladder cancer cells. **D**, **E** Representative images of EdU assay of bladder cancer cells. **F** MINDY1 depletion inhibits the tumor growth in vivo. T24 cells were stably transfected with lentivirus carrying a scrambled shRNA or MINDY1 shRNA. 1 × 10^6^ T24 cells were injected to the right dorsal flank of each mouse (n = 6). Tumor sizes were measured every 5 days until the end of the experiment. **G** Representative images of immunohistochemical staining for Ki67, MINDY1 and YAP. *, *P value* < 0.05; **, P value < 0.01; ***, P value < *0.001*

We next examined the role of MINDY1 in regulating bladder cancer proliferation. Our results demonstrated that depletion of MINDY1 significantly decreased cell proliferation and increased the population in G1 phases, indicating that MINDY1 may regulate G1 to S transition in bladder cancer cells (Fig. 5A, B). The results of clone formation assay revealed that MINDY1 depletion dramatically decreased the clone formation capability in T24 and UMUC3 cells (Fig. 5C). Consistently, EdU incorporation assay indicated that DNA synthesis was inhibited in T24 and UMUC3 cells treated with MINDY1 siRNAs (Fig. 5D, E). Then, we further investigated the role of MINDY1 in tumor growth by xenograft mice models. Our data showed that MINDY1 depletion by lentivirus-based shRNA decelerated bladder tumor growth (Fig. 5F, G).

### MINDY1 regulates cell proliferation through YAP

**Fig. 6:**
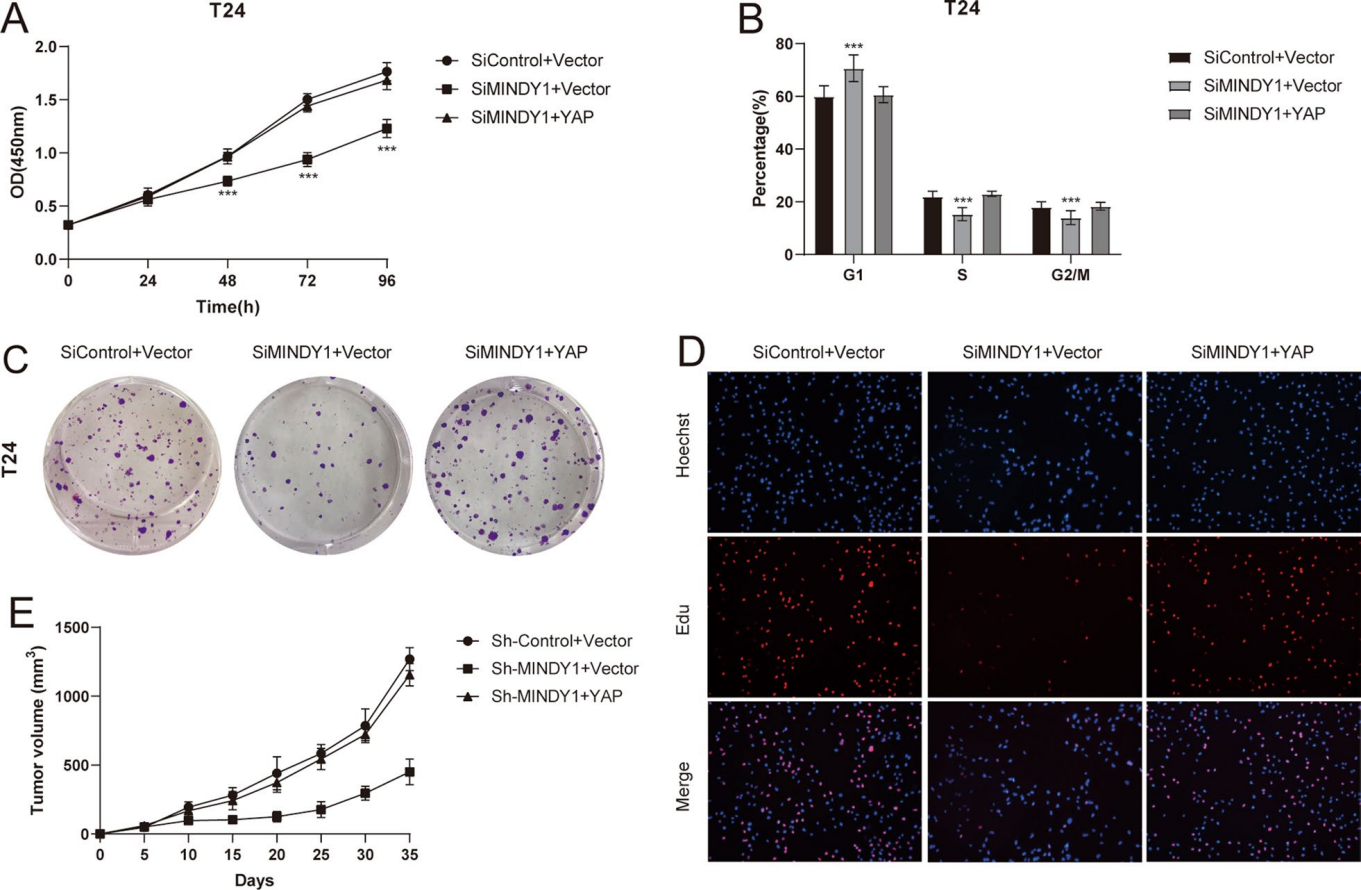
Increased YAP expression reverses the effect of MINDY1 depletion. **A** Cell proliferation assay of T24. **B** Cell cycle assay of T24. **C** Clone formation assay of T24. **D** Representative images of EdU assay of T24. **E** Overexpression of YAP in MINDY1-knockdown cells recovered tumor growth in vivo. *, *P value* < 0.05; **, P value < 0.01; ***, P value < *0.001*

To determine the mechanism of MINDY1 in regulating bladder cancer cell proliferation by stabilizing YAP, we performed rescue experiments by ectopic expressing YAP in MINDY1 knockdown T24 cells. CCK8 assay indicated that overexpression of YAP, largely recovered the proliferation ability and decreased the population of T24 cells in G1 phases (Fig. 6A, B). Increased YAP expression reversed the clone formation ability of T24 cells (Fig. 6C). Consistently, YAP overexpression also facilitated the DNA synthesis in T24 cells depleted with MINDY1 (Fig. 6D). Knockdown of MINDY1 significantly inhibited tumor growth in vivo, while the restoration of YAP expression abolished the inhibition induced by MINDY1 depletion (Fig. 6E). These results indicated that MINDY1 promoted bladder cancer cell proliferation, at least partially, via the regulation of YAP.

### MINDY1 and YAP are uniformly overexpressed in bladder cancer samples

**Fig. 7:**
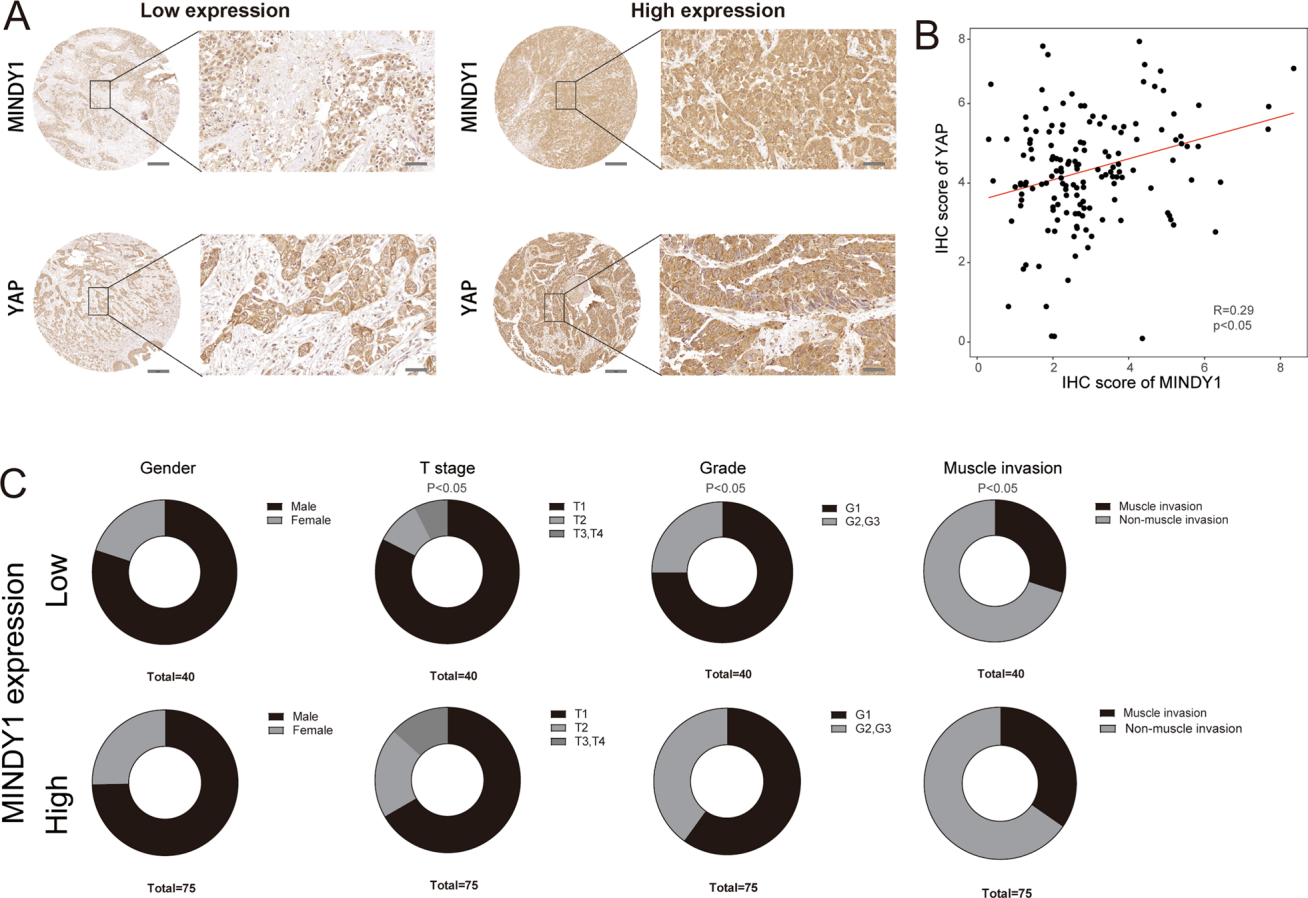
MINDY1 correlates with YAP protein levels in human bladder cancer samples. Tissue microarray was obtained from Alenabio Company Co., Ltd, Shanxi, China. The tissue microarray contained 115 bladder cancer specimens. **A** The typical staining of MINY1 and YAP in bladder cancer specimens. **B** YAP positively correlated with MINDY1 in bladder cancer samples (Pearson correlation). **C** MINDY1expression was associated with the tumor T stage, tumor grade and muscle invasion. The characteristics were compared between MINDY1 low-/high- groups using chi-square or Fisher’s exact tests

To further study the relationship of MINDY1 and YAP in bladder cancer, we examined the expression of MINDY1 and YAP in bladder cancer tissues using tissue microarrays (115 bladder cancer specimens). Immunohistochemistry staining showed a significant positive correlation between MINDY1 and YAP protein level in bladder cancer tissues (Fig. 7A, B). This positive correlation further proved the regulating relationship between MINDY1 and YAP. And as shown in Fig. 7C, MINDY1 expression was associated with tumor T stage, tumor grade, and muscle invasion in bladder cancer.

## Discussion

Bladder cancer is considered as one of the most frequently diagnosed and lethal human malignancy [20]. The therapeutic effect of conventional therapies is not satisfactory, patients who underwent radical cystectomy had a 5-year overall survival rate of 66% and a 10-year survival rate of 43% [21]. Recent advance in understanding the genetic and molecular mechanisms of bladder cancer hold promise for targeted therapy for this disease. Many signaling pathways are involved in the the survival of bladder cancer cells, including NF-kB, MAPK, mTOR and JAK-STAT [22]. Ubiquitination is an important posttranslational modification, which is a central component of the cellular protein-degradation machinery and essential for cellular homeostasis [23]. The major part of ubiquitination process is mediated by three enzymes: ubiquitin-activating enzyme (E1), a ubiquitin conjugating enzyme (E2) and a ubiquitin ligase (E3) [24]. It should be noted that the ubiquitination of cellular proteins is a reversible and dynamic process, constantly being ubiquitinated and deubiquitinated. This process is precisely and orchestrated determined by several E3 ubiquitin ligases and DUBs [25–27]. The E3 ubiquitin ligases selectively mediate the ubiquitin conjugation of substrates, while DUBs negatively regulate this process [28]. Accumulating evidence has confirmed that DUBs play an important role in cancer progression at multiple levels. Some DUBs, including UCHL1, BAP1 and CYLD, are described as displaying intrinsic oncogenic or tumor suppressor activities [29]. A number of DUBs, such as USP1, USP7 and UCHL5, have been indicated to regulate the levels or activities of various oncogene or tumor suppressor proteins through their deubiquitylating activities [30]. However, the potential roles of DUBs in bladder cancer are largely unknown.

The Hippo signaling pathway is a novel and evolutionary conserved pathway, and has emerged as a critical signaling in regulating tumorigenesis. The transcriptional coactivators TAZ and YAP are the final transducer effectors of this pathway, which interact with TEA domain family transcription factors to activate the transcription of genes involved in various oncogenic activities, including cell growth, cell mobility, cell survival and metabolism [8, 31]. Previous studies indicated that YAP was highly expressed in bladder cancer clinical samples and the expression level of YAP was associated with poor prognosis. YAP had crucial roles in the proliferation and migration of bladder cancer [32–35]. The activation of YAP is also associated the chemoresistance and radiation effects of bladder cancer. Overexpression of YAP protects while depletion of YAP sensitizes bladder cancer cells to chemotherapy through increasing the DNA damage and apoptosis [33]. The kinase cascade MST1/2-Lats1/2 directly phosphorylates YAP, which leads to cytoplasmic retention of phosphorylated YAP and consequentially results in ubiquitination and subsequent proteasomal degradation. The regulation of core components in the Hippo pathway by phosphorylation has been well investigated, but the roles of ubiquitination−deubiquitination processes remain largely unknown. ITCH is an E3 ubiquitin ligase that promotes the degradation of LATS kinases. A recent study reported that YOD1 could deubiquitinate ITCH, and enhanced the stability of ITCH, which subsequently reduced the levels of LATS and increased the YAP/TAZ level. YOD1 promoted the proliferation of hepatocytes and led to hepatomegaly depending on the YAP/TAZ activity [36]. OTUB2 was reported to enhance metastasis through Hippo-independent activation of YAP/TAZ signaling, which could stabilize YAP/TAZ by deubiquitination [37]. USP10 is a potent YAP/TAZ-activating DUB. It directly interacted with and stabilized YAP/TAZ by reverting their proteolytic ubiquitination. USP10 depletion enhanced the polyubiquitination of YAP/TAZ, promoted their proteasomal degradation, and ultimately arrested the proliferation of hepatocellular carcinoma in vitro and in vivo [38].

In the present study, we found that MINDY1 was a novel modulator in controlling YAP ubiquitination and stability, which depends on its DUB activity. Depletion of MINDY1 significantly decreased the YAP protein level and inhibited the Hippo signaling activity. The analysis of public available data based on TCGA indicated that MINDY1 was amplificated in bladder cancer. We observed an intimate correlation between MINDY1 expression and YAP protein level in human bladder cancer samples. We further explored the molecular mechanism of MINDY1 in regulating YAP, and found that YAP protein level was significantly decreased upon MINDY1 depletion. When cells were treated with the CHX, the half-life of YAP was significantly shortened in cells depleted of MINDY1, but prolonged in cells overexpressing the wild type MINDY1. We then tested whether ubiquitin–proteasome system (UPS) was required to YAP degradation induced by MINDY1 depletion, and found that MG132 largely recovered the decreased YAP expression induced by MINDY1 silence. We also identified that MINDY1 co-located with YAP. The Co-IP experiment demonstrated that endogenous MINDY1 coimmunoprecipitated with endogenous YAP. Deletion analysis demonstrated that the catalytic domain of MINDY1 physically interacted with the WW domain of YAP. The present study also demonstrated that MINDY1 removed the K48-linked ubiquitin chain from YAP, thus inhibiting proteasome-mediated YAP degradation. In addition, catalytically inactive mutant of MINDY1 (C137A) did not regulate the level of ubiquitination on YAP, suggesting that MINDY1 promoted-YAP stability was a consequence of the enzymatically active site of MINDY1 catalyzed-YAP deubiquitination. Our data further demonstrated that MINDY1 depletion dramatically decreased the proliferation bladder cancer cells. And the suppression effects induced by MINDY1 depletion could be reversed by YAP overexpression. These results demonstrated that MINDY1 promoted bladder cancer proliferation through increasing YAP stability. It is reported that targeting YAP and YAP-associated proteins may provide clinical benefit in bladder cancer patients [39, 40]. While lacking of efficient small molecule inhibitors for YAP currently make it difficult to target. No directly YAP/TAZ-targeting compounds have been identified so far [41, 42]. In addition, using small molecule inhibitors against YAP cannot completely abolish YAP transcriptional activity and is not very effective in treating YAP-driven cancers [43]. Therefore, targeting MINDY1 may also provide a new means to inhibit tumor‐specific YAP activity.

## Conclusions

**Fig. 8:**
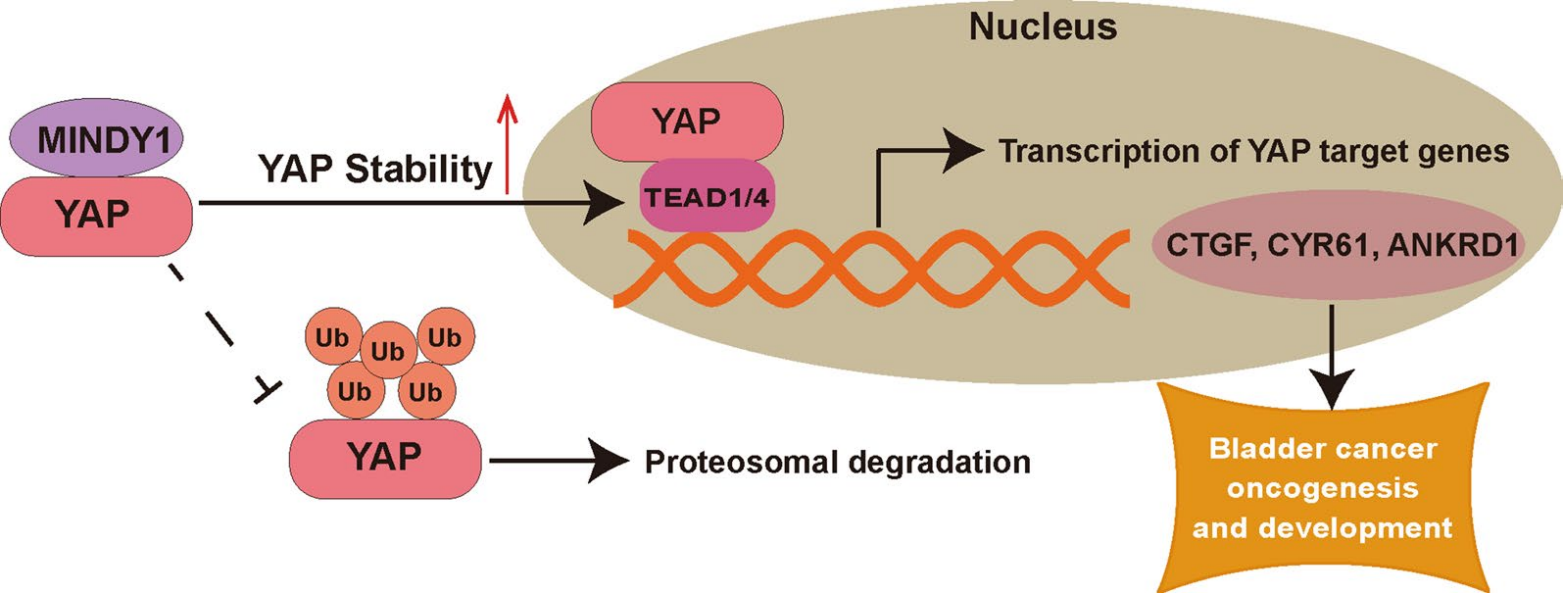
Mechanism diagram. Our study demonstrated that MINDY1 could promote bladder cancer cells proliferation in vitro and in vivo. Furthermore, MINDY1 could increase YAP protein stability by removing K48-linked Ub chains

In the present study, we examined the role of MINDY1 in bladder cancer cells and identified MINDY1 as the deubiquitinase to mediate YAP deubiquitination. MINDY1 was shown to associate with YAP protein and prolong its stability via removing the K48-linked ubiquitin chain from YAP (Fig. 8). Our data suggest that MINDY1 may drive bladder cancer progression via YAP expression. MINDY1 may be a potential target for bladder cancer intervention.

## Supplementary Information

**Additional file 1: Figure S1.** Western blot analysis of MINDY1 protein abundance in four bladder cancer cell lines (5637, UM-UC-3, T24 and BIU-87) and immortalized normal uroepithelial cell line (SV-HUC-1).

## Data Availability

Data sharing is not applicable to this article as no datasets were generated or analysed during the current study.
