# Supplementary figures and images for: MINDY1 promotes bladder cancer progression by stabilizing YAP
Source: Cancer Cell Int. 2021 Jul 27;21:395. doi: 10.1186/s12935-021-02095-4 (PMC8314533; doi:10.1186/s12935-021-02095-4)

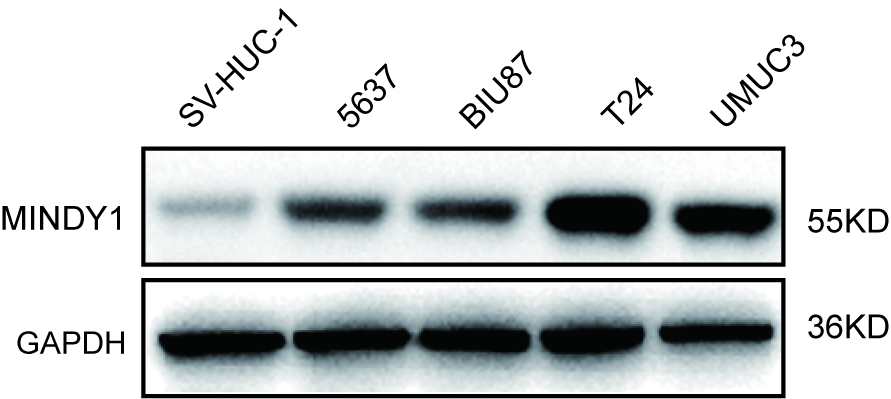

Supplement: Supplementary file 1 — Additional file 1: Figure S1. Western blot analysis of MINDY1 protein abundance in four bladder cancer cell lines (5637, UM-UC-3, T24 and BIU-87) and immortalized normal uroepithelial cell line (SV-HUC-1). [file 12935_2021_2095_MOESM1_ESM.tif]
